# An Appraisal of Human Mitochondrial DNA Instability: New Insights into the Role of Non-Canonical DNA Structures and Sequence Motifs

**DOI:** 10.1371/journal.pone.0059907

**Published:** 2013-03-29

**Authors:** Pedro H. Oliveira, Cláudia Lobato da Silva, Joaquim M. S. Cabral

**Affiliations:** Department of Bioengineering and Institute for Biotechnology and Bioengineering, Instituto Superior Técnico, Lisbon, Portugal; Fred Hutchinson Cancer Research Center, United States of America

## Abstract

Mitochondrial DNA (mtDNA) deletion mutations are frequently observed in aged postmitotic tissues and are the cause of a wide range of human disorders. Presently, the molecular bases underlying mtDNA deletion formation remain a matter of intense debate, and it is commonly accepted that several mechanisms contribute to the spectra of mutations in the mitochondrial genome. In this work we performed an extensive screening of human mtDNA deletions and evaluated the association between breakpoint density and presence of non-canonical DNA elements and over-represented sequence motifs. Our observations support the involvement of helix-distorting intrinsically curved regions and long G-tetrads in eliciting instability events. In addition, higher breakpoint densities were consistently observed within GC-skewed regions and in the close vicinity of the degenerate sequence motif YMMYMNNMMHM. A parallelism is also established with hot spot motifs previously identified in the nuclear genome, as well as with the minimal binding site for the mitochondrial transcription termination factor mTERF. This study extends the current knowledge on the mechanisms driving mitochondrial rearrangements and opens up exciting avenues for further research.

## Introduction

Rearrangement of mitochondrial DNA (mtDNA) and consequent loss of mitochondrial function has been implicated in the aging process and in a broad range of clinical phenotypes (reviewed in [1]). Short direct and inverted repeats are found to flank the majority of such rearrangements (∼85%), a fact that has led to the assumption that deletion formation arises from slipped mispairing during replication or repair of damaged DNA [2]. It appears, however, that no significant correlation exists between the density of repeat pairs and distribution of deletion breakpoints [3], suggesting that additional factors are likely to contribute to the mutational spectra. In human mtDNA rearrangements, the 5′ breakpoints have a typical unimodal distribution with a maximum around 8 to 9 kb, whereas the 3′ breakpoints are preferentially clustered above 13 kb [3]. This distribution bias has led Samuels and co-workers [3] to present evidence strongly favoring the existence of a unique and similar mechanism involved in the formation of all mtDNA deletions, irrespective of their immediate deletion breakpoints or presence of repeated sequences. The authors show that the 13 bp direct repeats responsible for the 4,977 bp “common deletion”, lie in the center of the distribution of other deletions, and are therefore at least partially responsible for governing the latter. Later on, an elegant computational analysis by Guo and colleagues [4] has refuted the bimodal hypothesis proposed in Samuels’s work, by essentially showing that the distribution of deletions in individuals lacking the 13 bp repeat pair is roughly the same as those found in control samples containing it. In alternative, they suggest that deletions arise preferentially through the formation of distant segments of mtDNA capable of forming stable imperfect duplexes. According to this view, after the duplex is formed, the deletion will occur at one of the many perfect repeats available nearby. Further evidence point towards an evolutionary selection pressure against long and stable repeats in long living mammals such as the human, but not in short-lived ones [5], [6].

More recently, Damas and co-workers [7] have found that the most frequent deletion breakpoints occur within or near regions showing non-canonical (non-B) conformations, namely hairpins, cruciforms and cloverleaf-like elements. These important findings are in line with two earlier studies that support non-B-mediated instability: the first, published more than a decade ago, found that bent-inducing sequences render certain regions of the mitochondrial genome more labile to attack by reactive oxygen species or more prone to undergo deletions or duplications [8], [9]. The second observed that several direct repeats flanking mtDNA rearrangements have a skewed base composition rich in pyrimidines at the level of the light strand, thus suggesting the formation of a triple-helix structure between repeats [10].

When considering all of the above, it becomes clearer that none of the theories proposed so far is broad enough to explain all variants of the mutational spectra. Instead, they collectively point for a multifactorial view of human mtDNA instability, as previously suggested [4]. In this work we started by evaluating the importance of alternative non-canonical structures (e.g. intrinsically curved DNA, G-quadruplexes, triplex DNA and Z-DNA), whose impact on mtDNA instability is unknown or has so far been poorly explored in the literature. Most interestingly, our analysis revealed that the biased distribution of breakpoints along the mitochondrial genome correlates significantly with local compositional skews and with the presence of a degenerate sequence motif whose biological significance is discussed. In combination with past studies, the data shown here may help to understand and redefine the multiple mechanisms by which deletion formation occurs in the human mitochondrial genome.

## Methods

### Wild-type Human Mitochondrial DNA Sequence

The light strand of the mitochondrial revised Cambridge reference sequence (rCRS, accession number: NC_012920) was used throughout this study.

### Meta-analysis of Deletion Breakpoints in Human mtDNA

We compiled 754 different mitochondrial deletions available at the Mitomap database (http://www.mitomap.org) and in published literature describing pathological and non-pathological clinical situations (see Table S1). These breakpoints were defined as the positions upstream of the 5′ break and downstream of the 3′ break and numbered according to the L-strand positions of the rCRS mtDNA sequence. During the breakpoint selection process, we have excluded breakpoint pairs in which both extremities were repeated. However, those deletions sharing one breakpoint and differing in another were considered as distinct, and were therefore included. Due to the presence of flanking repeats, intervals of values are often provided in the literature instead of the exact breakpoint positions (e.g. 7,508–7,515; 15,939–15,946). In these cases, we have maintained the smallest value for each breakpoint in the interval (7,508–15,939 in the latter example). This allowed for an easier comparison between our data and that provided by earlier reports [7]. The mutational spectra encompassing all of the 5′ and 3′ deletion breakpoints were plotted using R (http://cran.r-project.org).

### Curvature/Bendability Profiles and Three-dimensional Representation of DNA Sequences

Curvature propensity plots were obtained using the BEND algorithm [11] by submission of DNA sequences to the bend.it server (http://hydra.icgeb.trieste.it/dna/bend_it.html) [12] using the DNAse I-based parameters of [13]. This server calculates DNA curvature as a vector sum of dinucleotide geometries (roll, tilt and twist angles) and expresses it as degrees per helical turn (10.5°/helical turn = 1°/bp). DNA sequences were submitted in raw format and the predicted curvature and bendability were collected by E-mail in ASCII format. Three-dimensional representation of the curvature profiles was performed with the model.it server (http://hydra.icgeb.trieste.it/dna/model_it.html) [12] and the output was displayed and visualized with MOLEGRO Molecular Viewer (http://www.molegro.com/mmv-product.php).

### Sliding Window Analysis of GC-content and GC-skew

We have used the DNA base composition analysis tool (http://molbiol-tools.ca/Jie_Zheng/dna.html) to evaluate GC-content and GC-skew along the human mitochondrial genome using non-overlapping 20 bp sliding windows. GC-skew was calculated as (G-C)/(G+C).

### Randomization of Breakpoint Positions

For breakpoint randomization we have generated 200 datasets using two approaches. In the so-called *random* approach, 1,508 arbitrary deletion breakpoints (twice the number of deletions) were randomly distributed throughout the mitochondrial genome with no restrictions. In the *partially random* approach the same number of breakpoints was generated while maintaining their original abundance within the mitochondrial arcs and origins of replication. In both cases, repeated events were allowed to occur. In order to assess the significance of a given variable *P* we computed a *z*-score as:
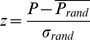
where 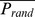 is the average of the randomized variable *P* and 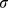 represents its standard deviation. The corresponding *p* values were obtained from 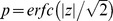, where *erfc* is the complement error function.

### *De novo* Motif Finding, Selection and Validation

Identification of G-quadruplex structures was performed with the Quadparser algorithm [14] by searching for sequences complying with the canonical folding rule G≥_3_N_1–7_G≥_3_N_1–7_G≥_3_N_1–7_G≥_3_ and C≥_3_N_1–7_C≥_3_N_1–7_C≥_3_N_1–7_C≥_3_. It should be noted that Quadparser outputs only distinctive and non-overlapping sequences, irrespectively of the number of G or C runs present in the motif. Also, if runs of different length coexist in the same motif, more than one topological rearrangement could occur, and in this case, Quadparser will again output it as a single site. Search for triplex DNA and Z-DNA motifs was performed with the non-B DNA motif search tool (nBMST) [15]. For *de novo* search of over-represented motifs in the vicinity of the breakpoint dataset, we started by extracting all the ±15 bp flanking regions using the window extractor DNA feature of the Sequence Manipulation Suite (http://www.bioinformatics.org/sms2/window_extract_dna.html). Two observations should be made on this: first, the length chosen for the flanking windows results from a compromise between the more common range of motif lengths (6–12 nt) and the need to minimize the chance of getting false positives. Even so, similar results were observed when conducting a motif search considering ±25 bp flanking regions and motif lengths between 6–20 nt (data not shown). Second, data extraction was performed using the non-repeated breakpoint dataset to avoid biasing the results. Motif search was performed with the Multiple Expectation Maximization for Motif Elicitation tool (MEME) [16] and the Gibbs sampling algorithm AlignACE [17]. MEME search was carried out to detect motifs of length 6–20 nt, using both 'zoops' (zero or one occurrence per sequence) and 'anr' (any number of repetitions) options. In AlignACE, the background GC-content parameter ('gcback') was set to 0.471, which corresponds to the fractional GC-content of the breakpoint regions. Moreover, the number of columns to align ('numcols' parameter) was set to 6–20 nt and the expected number of motifs to find ('expect' parameter) was arbitrarily set to 3. From the output file, only motifs with maximum *a priori* log likelihood (MAP) scores higher than 200 were accepted, since this value is above the typical ranges considered as biologically significant (usually above 10). Each motif set obtained was then visually compared, and only those consistently and simultaneously predicted by both algorithms were considered as strong. Closely related motif groups identified by the same program were discarded. Motifs consisting of single nucleotide repeats of the type P_n_ were manually parsed out, irrespectively of their positions or number of occurrences. For consensus analysis, the position-specific probability matrix (PSPM)-derived motifs were plotted with Weblogo [18]. To confirm that the similar motif sets were properly grouped, we used the web-tool STAMP [19], which allowed motif edge trimming whenever the information content was below 0.4. To statistically validate the significance of the motifs found, we have calculated its background occurrence by randomly shuffling the mitochondrial genome 130 times preserving k-tuples of length 1 and 3, respectively using the shuffleseq tool from the EMBOSS suite [20] and the gshuf program (kindly provided by Eduardo Rocha from Institut Pasteur). Moreover, the physical distances between each breakpoint and the closest motif were computed in both the mitochondrial genome and shuffled data sets. Statistical significance (*p*-values) was calculated from corresponding *z*-scores.

## Results

### Deletion Breakpoints are Non-randomly Distributed in the Human Mitochondrial Genome

**Figure 1:**
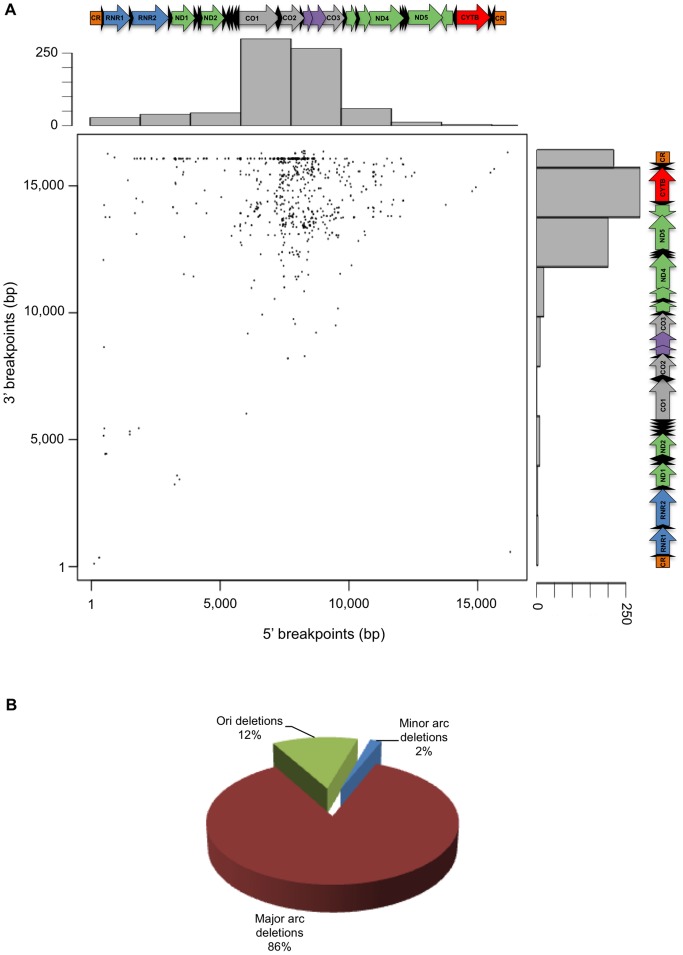
Human mitochondrial deletion spectra. (A) Distribution of the 5′ and 3′ positions corresponding to 1,508 breakpoints, as well as corresponding histograms and positions along the mitochondrial genome. CR-control region; RNR-Ribosomal RNA; ND-NADH dehydrogenase; CO-cytochrome oxidase; CYTB-Cytochrome B. Black arrows correspond to the 22 tRNA genes. (B) Pie chart indicating the proportion of deletions occurring exclusively in the major arc (nucleotide positions 1–109 and 5,799–16,569), minor arc (nucleotide positions 442–5,720) or involving the origins of replication O_H_ (nucleotide positions 110–441 bp) and O_L_ (nucleotide positions 5,721–5,798).

The set of 754 deletions gathered in this work, has only minor differences to that recently published in [7] (see Table S1). Among the 1,508 deletion breakpoints (twice the number of deletions), 1,115 were found to be different. There is a clear preference for 5′ breakpoints to map in the vicinity of position 7.7 kb and 3′ breakpoints to map in the vicinity of positions 14.5 and 16.1 kb (Fig. 1A). The latter positions fall within or around the *CO2* gene (5′ breakpoints) and *ND6* and *CYTB* genes (3′ breakpoints). Well-known examples of deletion hot spots located in the close vicinity of these positions are the “common deletion” (nucleotide positions 5′ 8,470–8,482; 3′ 13,447–13,459) or the displacement loop (D-loop) 16,070 regions. The large majority of human mtDNA deletions (86%) affect solely the major arc (nucleotide positions 5,799–16,569 and 1–109), 2% affect the minor arc (nucleotide positions 442–5,720) and 12% affect the origins of replication (nucleotide positions 110–441 and 5,721–5,798 respectively for O_H_ and O_L_) (Fig. 1B). The average global density of breakpoints (per 0.1 kb) is 9.1, whereas partial densities in the minor arc and major arc are respectively 1.8 and 12.7. Minor arc deletions are typically smaller, harder to detect and not as widely associated with disease phenotypes as those found in the major arc. This fact may contribute to the disparity of available data between arcs. Moreover, there is a general consensus that the strand-asynchronous asymmetric replication mode of the mtDNA favors the occurrence of aberrations within the major arc. As pointed out before [7], this non-stochastic distribution of deletion breakpoints departs significantly from that obtained in a non-restricted simulated random model, which reinforces the idea that certain drivers of instability might be over-represented in the abovementioned regions.

### Breakpoints are Preferentially Clustered in the Close Vicinity of Intrinsically Curved Regions

**Figure 2:**
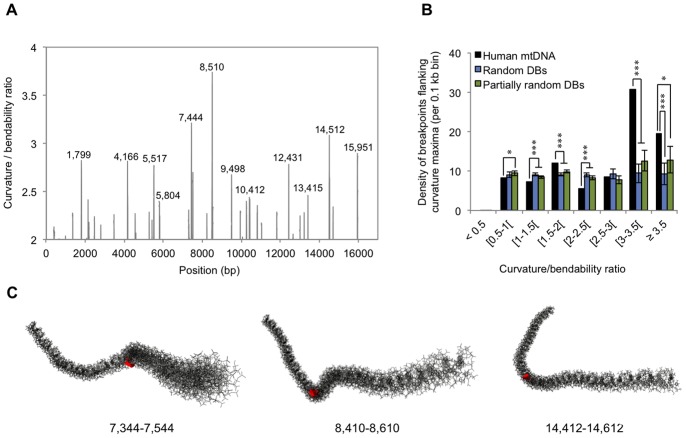
Impact of intrinsically bent DNA in the distribution of deletion breakpoints. (A) Curvature/bendability profile of the entire mtDNA genome as computed by the bend.it algorithm (see Methods section). The exact positions corresponding to the highest curvature/bendability ratios are indicated above the corresponding peaks. (B) Density of deletion breakpoints (∑ number of breakpoints/∑ fragment sizes) computed in 0.1 kb bins flanking each curvature maximum (black bars). Also shown are the density values computed after randomization of breakpoint positions (shown in blue) or after partially randomization of breakpoint positions (shown in green) (see also Methods section). (C) Three-dimensional representations of three 0.2 kb regions harboring highly curved sequences. The exact position corresponding to each curvature maximum is highlighted in red. Additional three-dimensional representations of the remaining peaks highlighted in (A) can be found as supplementary material (Fig. S1). Error bars represent standard deviations. * *p*<0.05; *** *p*<0.001.

It was recently shown that intra-strand DNA hairpins and cloverleaf-like elements are enriched in common breakpoint sites of the human and mouse mitochondrial genomes [7]. These observations prompted us to investigate if breakpoints were preferentially located within or in the close vicinity of other classes of non-B DNA elements. The intrinsic flexibility of a DNA molecule (bendability) and its tendency to form a bent structure in the absence of external forces (curvature propensity) are parameters commonly used to describe secondary structure. A highly bendable molecule is less rigid, and does not necessarily retain intrinsic curvature as it allows a mixture of many different conformational states [21]. Thus, regions having high curvature/bendability ratios are more prone to adopt curved and rigid conformations with elevated topological stress. In this sense, we decided to evaluate if human mitochondrial deletion breakpoints were preferentially clustered in regions under high torsional stress, and if known hotspots such as the “common deletion” are located in regions with particularly high ratios. While analyzing the mtDNA curvature/bendability profile (Figure 2A), we observed that the locations of the highest peaks (nucleotide positions 7,444; 8,510; 14,512; 15,951) fall within regions of high breakpoint density (compare with Fig. 1A). In particular, the 8,510 peak closely matches the 5′ breakpoint of the “common deletion”, and the 15,951 peak locates in the vicinity of the 16,070 hotspot. Given the fact that the curvature/bendability ratio can change abruptly in just a few base pairs, we hypothesized that a considerable number of breakpoint positions may have a low ratio but still locate in the vicinity (±50 bp) of a curvature maximum. To evaluate the possibility for such distribution bias, we considered all breakpoints mapping in 0.1 kb bins centered in each local maximum of the mitochondrial genome and computed their corresponding densities (Fig. 2B). The highest breakpoint densities were found in those regions with the highest curvature/bendability ratios, and departed significantly from density values estimated to occur randomly (Fig. 2B). These high breakpoint density values were found within the *CO1*, *tRNA* Ser, *ATPase8* and *ND6* genes. Despite the generalized decrease in breakpoint density observed at ratios below 3, 90.5% of all breakpoints locate in the close vicinity of regions with ratios above the average value for the human mitochondrial genome (0.85). The increased topological stress of regions harboring high breakpoint density becomes more obvious after inspection of their three-dimensional structure. Fig. 1C depicts the three regions with the highest ratios in the genome, whereas the remaining regions are shown as supplementary material (Fig. S1). The adoption of S- or elbow-like structures with low flexibility might contribute to an increased frequency of genetic instability events, likely due to replication fork stalling or increased susceptibility to reactive oxygen species.

### Large G-quadruplexes are Enriched in Deletion Breakpoints

**Figure 3:**
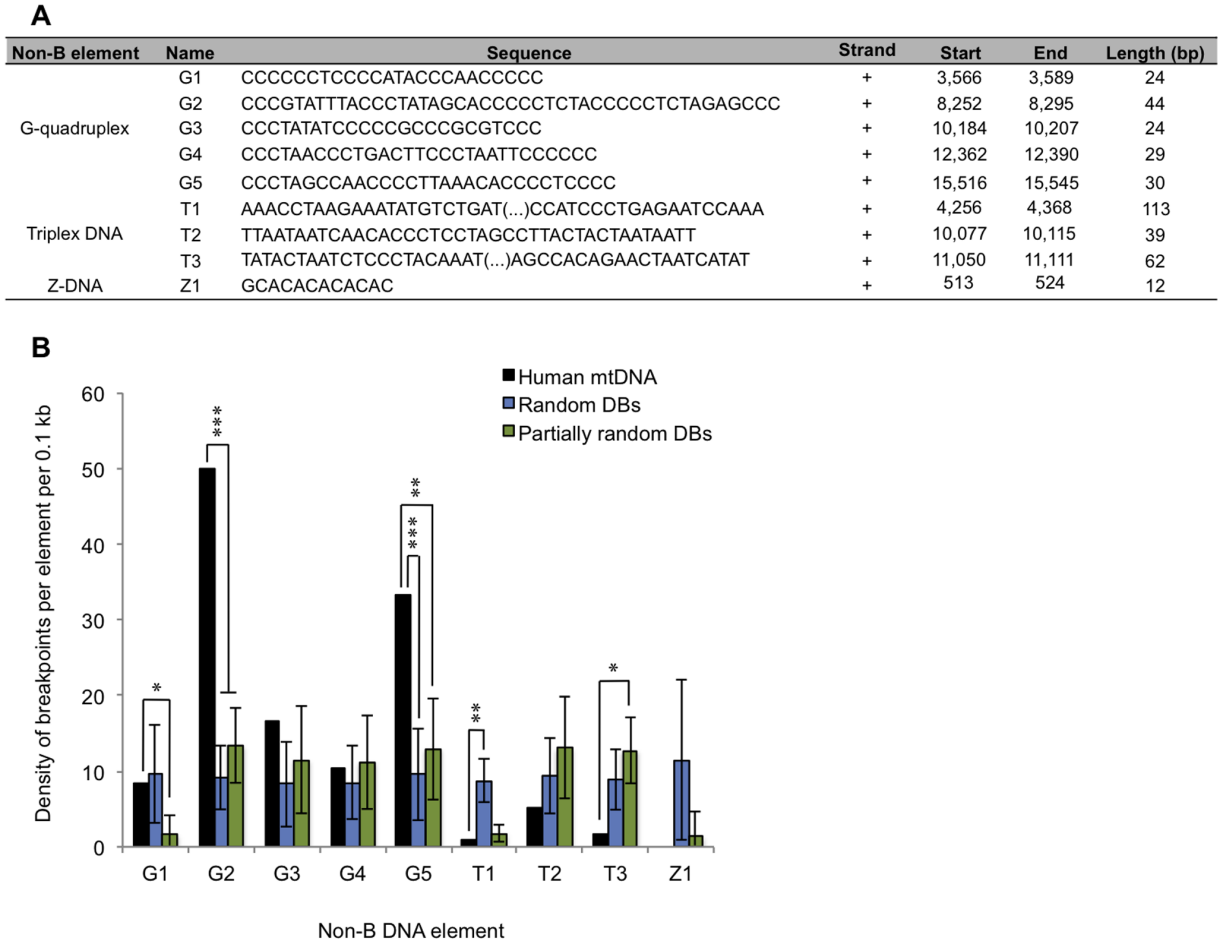
Impact of the presence of non-canonical (non-B) DNA structures on the distribution of deletion breakpoints. (A) Sequences and locations of the DNA strings predicted to fold into G-quadruplexes, triplexes and Z-DNA. (B) Density of deletion breakpoints per 0.1 kb bins of each non-B element (black bars). Also shown are the density values computed after randomization of breakpoint positions (shown in blue) or after partially randomization of breakpoint positions (shown in green). Error bars represent standard deviations. * *p*<0.05; ** *p*<0.01; *** *p*<0.001.

Our previous observations prompted us to pinpoint additional non-B structures within the human mtDNA, particularly sequences capable of forming G-quadruplexes, triplex DNA and Z-DNA, and evaluate their enrichment in deletion breakpoints. A burgeoning body of evidence supports the involvement of such structures in genomic instability events [22]–[24], but their role in the human mitochondrial genome has not been thoroughly explored. Both the Quadparser and nBMST tools were used to search for sequences that can potentially fold into such structures (see Methods for more details). We have found five G-tetrads, three sequences prone to generate triplex DNA and one sequence prone to generate Z-DNA (Fig. 3A). The local average density of breakpoints found in these nine sequences was 14.0 per 0.1 kb, which corresponds to a fold increase of 1.5 when compared to the average for the mitochondrial genome. When we compared the real breakpoint densities within these structures with those predicted from the random and partially random models, we verified that only the G2 and G5 elements were significantly enriched in breakpoints (Fig. 3B). The average breakpoint density found for these two elements was 41.7 per 0.1 kb, which corresponds to a fold increase of 4.6 and 3.3 when respectively compared to the genome average and major arc densities. Interestingly, G2 and G5 show the largest sizes among their class (Fig. 3A). In view of these observations, it is plausible to speculate that the presence of G-quadruplexes above a certain threshold size may generate more stable and bulkier structures capable of causing replication fork stalling. Apart from carrying a local over-representation of deletion breakpoints, G2 and G5 are also located in the close vicinity of regions displaying the highest frequency of instability events (compare Fig. 3A and Fig. 1A). G2 maps within the *CO2* and *tRNA* Lys genes whereas G5 maps within the *CYTB* gene. Concerning triplex DNA, it does not seem to play an influential role in promoting mitochondrial instability, since the number of breakpoints found was generally under-represented when compared to the random and partially-random models, even in large elements such as T1 (Fig. 3B). No deletion breakpoints were found within the Z-DNA element (Fig. 3B), which was expected since mutations occurring in the D-loop region tend to be strongly selected against due to their potential effect on replication and copy number.

### Deletion Breakpoints are Over-represented in GC-skewed Regions and in the Close Vicinity of a Degenerate Sequence Motif

**Figure 4:**
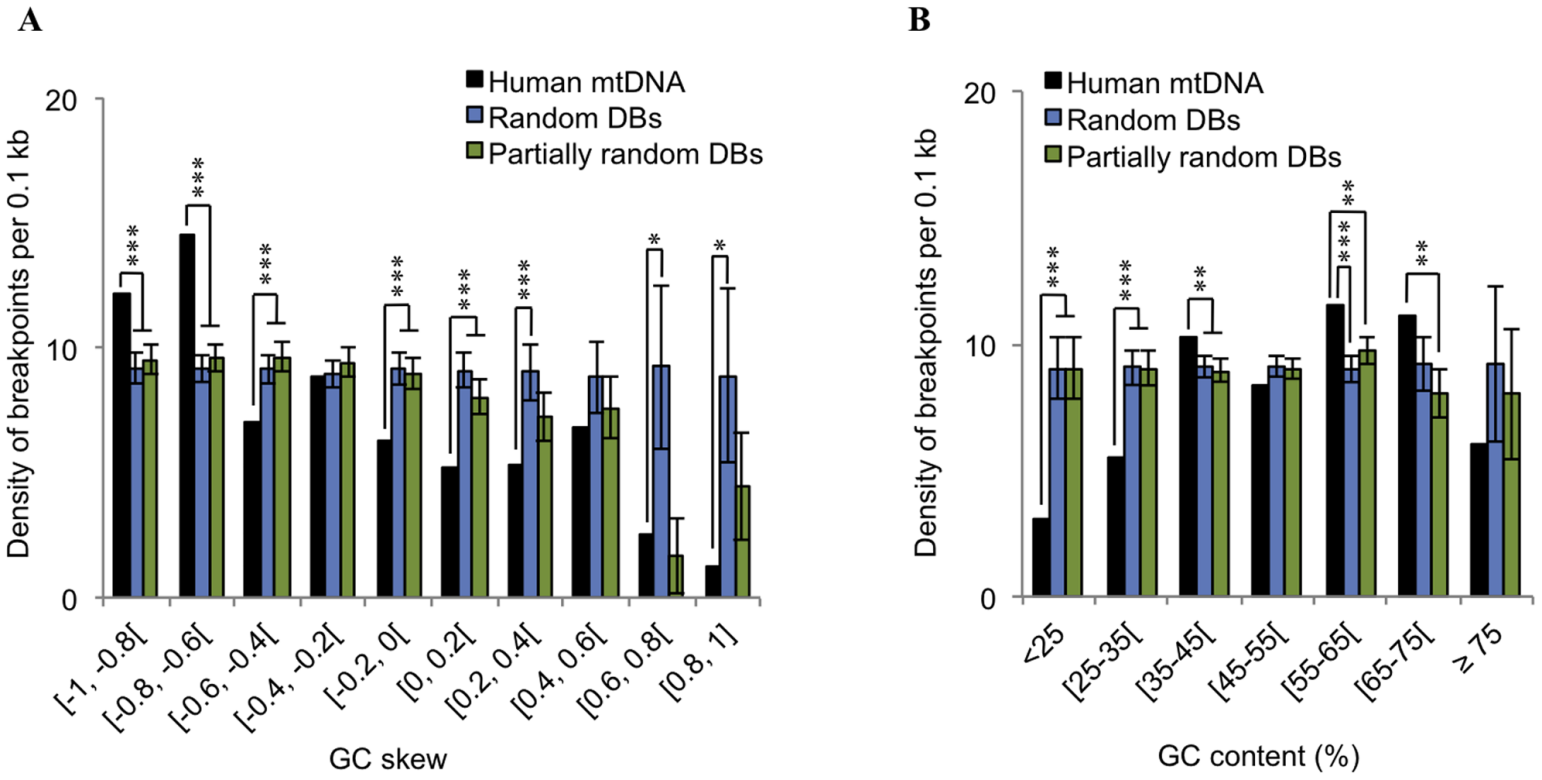
Impact of GC-skew and % GC-content in the distribution of deletion breakpoints. Variation in the density of deletion breakpoints *per* 0.1 kb with GC-skew (A) and % GC-content (B). Black bars represent the density values obtained for the human mtDNA, whereas blue and green bars respectively represent the values computed after randomization and partial randomization of breakpoint positions. Error bars represent standard deviations. * *p*<0.05; ** *p*<0.01; *** *p*<0.001.

During our analysis, we frequently observed a GC- and, to a lesser extent, an AT-rich DNA context next to deletion breakpoints, a fact that is consistent with the formation of several non-B structures (reviewed in [23]). The particularly high density of breakpoints observed in such a small and compositionally biased portion of the genome (G-quadruplexes) prompted us to further investigate if mtDNA instability events could be concurrent to regions showing variations in GC-skew or GC-content. Both GC-content and GC-skew were measured in non-overlapping 20 bp sliding windows along the human mtDNA. The average % GC-content was found to be 44.4% while the average GC-skew was −0.41, due to the predominance of cytosine (and adenine) residues in the light strand [25]. In line with our finding that breakpoints were over-represented in G2 and G5, we also found a significant percentage of breakpoints (64%) mapping in regions in which GC-skew is below the genome average (Fig. 4A). In particular, regions having a GC-skew below −0.6, show the highest density of deletion breakpoints (above 12 per 0.1 kb), significantly departing from the values expected to occur by chance (Fig. 4A). On the other hand, roughly 60% of all breakpoints are located in regions with a % GC-content above the genome average (Fig. 4B). Concomitantly, breakpoints located in regions having a % GC-content between [55–75[ were found to be over-represented when comparing to the random and partially random models (Fig. 4B).

**Figure 5:**
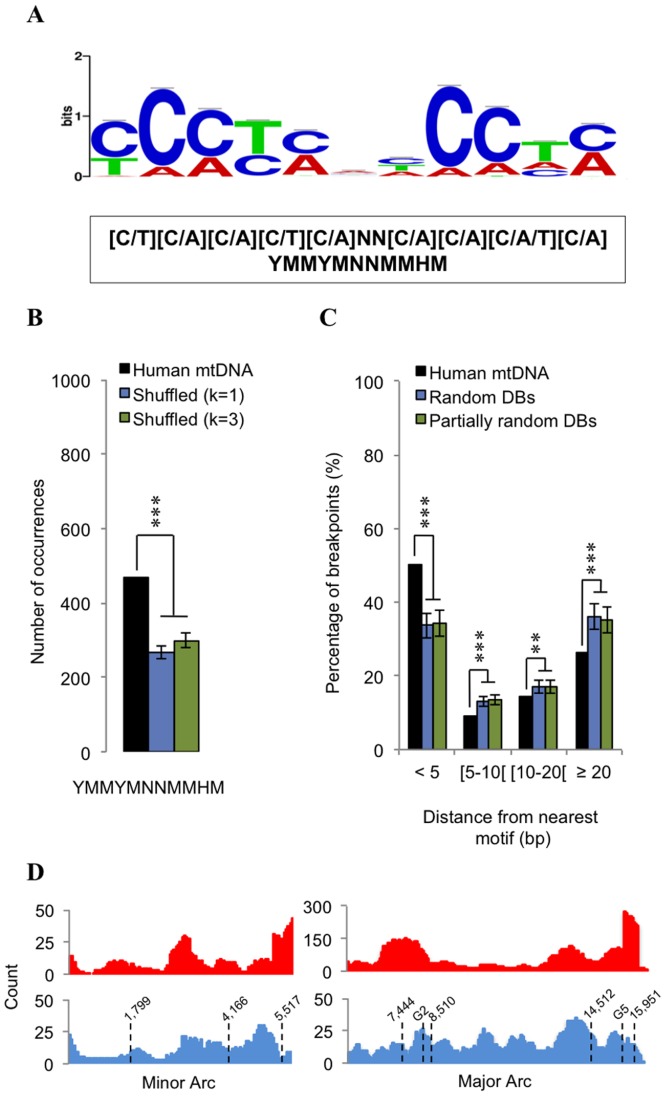
Search for over-represented motifs in the close vicinity of deletion breakpoints. (A) Sequence logo of the degenerate 11-mer motif over-represented in the close vicinity (±15 bp) of the non-repeated breakpoint dataset. Representative logos were obtained from MEME and AlignACE (see Fig. S2), and compared both manually and using the STAMP tool. Degenerate nucleotides are as follows: Y = (C or T); M = (A or C); H = (A or T or C); N = (A or T or G or C). (B) The number of occurrences of the YMMYMNNMMHM motif in the human mtDNA (black bar) is compared with those obtained from randomly shuffled genomes preserving k-tuples of 1 and 3 (respectively blue and green bars). (C) Percentage of breakpoints in terms of distance (bp) to the nearest YMMYMNNMMHM motif. Black bars represent the percentage values obtained for the human mtDNA, whereas blue and green bars respectively represent the values computed after randomization and partial randomization of deletion breakpoints. Error bars represent standard deviations. * *p*<0.05; ** *p*<0.01; *** *p*<0.001. (D) Distribution profiles of breakpoints (red) and YMMYMNNMMHM motif (blue) along the minor arc (left) and major arc (right). Stippled lines indicate the positions of the previously identified highly bent regions as well as of the G2 and G5 motifs.

These observations on the presence of compositional asymmetries near unstable regions are not only in line with our previous findings of non-B elements, but together with literature evidence (see Discussion section below), raise the possibility for the presence of other over-represented motifs. To evaluate this scenario, we carried out a search for conserved motifs in the close vicinity (±15 bp) of our non-repeated breakpoint dataset (n = 1,115). For this purpose, as well as to attain more reliable conclusions on over-represented motifs, we have used two different motif discovery tools, MEME and AlignAce, followed by motif edge trimming using STAMP (see Methods section for further details). An 11–mer degenerate consensus [C/T][C/A][C/A][C/T][C/A]NN[C/A][C/A][C/A/T][C/A] (or alternatively YMMYMNNMMHM) was found to be over-represented in our dataset (Fig. 5A and Fig. S2). This motif occurs 469 times in the human mtDNA, and was found to be over-represented when compared to shuffled mitochondrial genomes (Fig. 5B). 50.3% of all mtDNA breakpoints were observed at a distance of less than 5 bp from one of such motifs (Fig. 5C). This percentage increased to 73.7% when we considered a maximum breakpoint-motif distance of 20 bp (Fig. 5C). Also, the distribution of this motif was globally well correlated with the distribution of deletion breakpoints in both arcs (Spearman *ρ* = 0.38; *p*<0.001) (Fig. 5D). Still, some regions depart from this tendency and show extremely high counts of breakpoints, despite a weak increase in the number of YMMYMNNMMHM motifs (e.g. nucleotide positions position 5.5 in the minor arc and 7.5 and 16 kb in the major arc) (Fig. 5D). Bearing in mind our findings on intrinsic curvature, these local discrepancies correlate with the nearby presence of highly bent regions at positions 5,517, 7,444 and 15,951, which as we mentioned previously, likely play a destabilizing role in these regions.

## Discussion

It is now commonly accepted that the generation of large-scale mtDNA deletions can be attributed either to the formation of slipped structures during DNA replication, or alternatively, to the repair of strand breaks originated by fork stalling or ionizing radiation (reviewed in [26]). Although the causes behind fork arrest in the mitochondrial genome can be numerous, those that can be attributed to the formation of higher-order DNA structures, have only recently been given a predominant role in mitochondrial deletion formation [7]. The authors of this study point out the importance of non-B elements such as hairpins, cruciforms and cloverleaf-like elements in eliciting human mitochondrial DNA rearrangements either by facilitating fork arrest or nucleolytic attack. Building on this information, we examined whether additional DNA architectures could similarly impact the stability of the human mitochondrial genome. Our analysis revealed that intrinsically curved regions as well as large G-quadruplexes are enriched in deletion breakpoints. Bent DNA typically arises from the presence of short runs of regularly phased adenine:thymine tracts (helical periodicity of 10–11 bp), and its presence has been implicated in functionally relevant cellular processes including transcription [27], replication [28], and recombination [29], [30]. It has also been suggested that bent DNA might serve as recognition motif to the binding of topoisomerases and nucleases, thus facilitating breakage and subsequent illegitimate recombination or attack by reactive oxygen species [8], [9]. In a former study, the mobility of PCR-amplified and digested fragments of human mtDNA was evaluated by two-dimensional gel electrophoresis [9]. The authors found evidence for the presence of bent-like DNA, locating near or within deletion-prone regions (nucleotide positions 5,221–5,988, 6,928–7,493, 7,901–8,732 and 15,327–16,228). In our analysis we were able to narrow down these large regions to four peaks, respectively mapping at positions 5,517, 7,444, 8,510 and 15,951 (Fig. 2A). Together with the peak predicted at nucleotide position 14,512, these locations were found to concentrate in their ±50 bp vicinity, some of the highest breakpoint densities seen in the human mitochondrial genome (see Fig. 2A and 2B). We considered that deletion breakpoints might arise in poorly bent regions, but still as a consequence (or under partial influence) of the topological distortion induced by the presence of nearby curvature/bendability maxima. And despite the fact that this “proximity” effect becomes more obvious at highly distorted regions such as those mentioned above, we found that 90.6% of all breakpoints actually locate in the close vicinity of regions with curvature/bendability ratios above the average value found for the human mitochondrial genome (0.85).

In this work we also found an over-representation of deletion breakpoints in two large G-quadruplexes located at nucleotide positions 8,252–8,295 and 15,516–15,545. Such findings are consistent with the fact that quadruplexes are fork pausing sites capable of promoting recombination *in vitro* [31] and also over-represented in human recombination hotspots [22]. Although the detection of G-quadruplexes was made using the rule commonly associated with its canonical form (see Methods section), we do not discard the possibility that progenitor or degenerate forms eventually present (e.g. having different G runs or loop sizes) can also impact the stability of the human mtDNA.

The observation that mitochondrial deletion breakpoints were over-represented in negatively GC-skewed regions (Fig. 4A), prompted us to investigate the possibility of the presence of nearby hot motifs. This decision was further supported on the basis of three lines of evidence, according to which, distinct compositionally skewed motifs have been implicated in mitochondrial instability. The first line of evidence, refers to an intriguing finding brought to light in a recent study, in which was found that 12 out of the 13 bp direct repeats of the “common deletion” perfectly match a degenerate consensus motif (CCNCCNTNNCCNC) strongly over-represented in human nuclear recombination hot spots [32]. The latter, as well as the 9-mer CCCCACCCC were found to be implicated in allelic crossover activity during meiosis, nonallelic homologous recombination, and instability at hypervariable human minisatellites [32]. By also finding the presence of such motifs associated with the mitochondrial “common deletion”, the authors suggest in the same study, their implication in repeat-associated rearrangements, for example, by stimulating the formation of double-stranded breaks. A second line of evidence comes from a previous study on the mitochondrial transcription terminator factor mTERF, where it was shown that its binding sites (minimal consensus CCN_8_CC) in the human mitochondrial DNA are also replication pausing sites, which match frequent breakpoints in rearranged mtDNA “sublimons” [33]. The third line of evidence comes from the observation that poly(C) motifs such as CCTC and ACCC found in the D-loop hypervariable segment I and NADH dehydrogenase genes, are associated with a higher rate of point substitutions, small deletions and duplications [34], [35]. Also, local recombination rates were found to be positively correlated with GC-content across several human chromosomes [36], presumably resulting from non-adaptive processes such as GC-biased gene conversion [37]. Our search returned a unique highly degenerate motif, YMMYMNNMMHM, over-represented in the close vicinity (±15 bp) of our breakpoint dataset. Occurrences of this 11-mer motif match the positions of CCNCCNTNNCCNC flanking the common deletion, as well as one out of two occurrences of the CCCCACCCC motif. Its frequency distribution along the human mtDNA also correlates with that of the minimal mTERF binding site (Spearman *ρ*  = 0.58; *p*<0.001). We therefore anticipate a biological role for the YMMYMNNMMHM motif in eliciting instability events. One hypothesis is that its homopolymeric runs are capable of leading to an intracellular local depletion of a particular nucleotide, ultimately resulting in replication fork stalling followed by double strand break (DSB) formation [38]. In fact, replication stalling at homopolymeric runs has been previously pointed out as primary cause of mtDNA deletion formation in patients with autosomal dominant progressive external opthalmoplegia (adPEO) [38]. Another possibility is that the presence of the YMMYMNNMMHM motif somehow stimulates the formation of DNA•RNA hybrids (R-Loops) during replication, leading to genomic instability. Such structures have been shown to form preferentially at regions populated by short iterated repeats with a high GC-content, and to block the progression of the replication fork (reviewed in [39]). R-loops are known to form during the initiation of mammalian mtDNA replication [40], but in the last few years have been recurrently linked to DNA instability phenomena [41].

**Figure 6:**
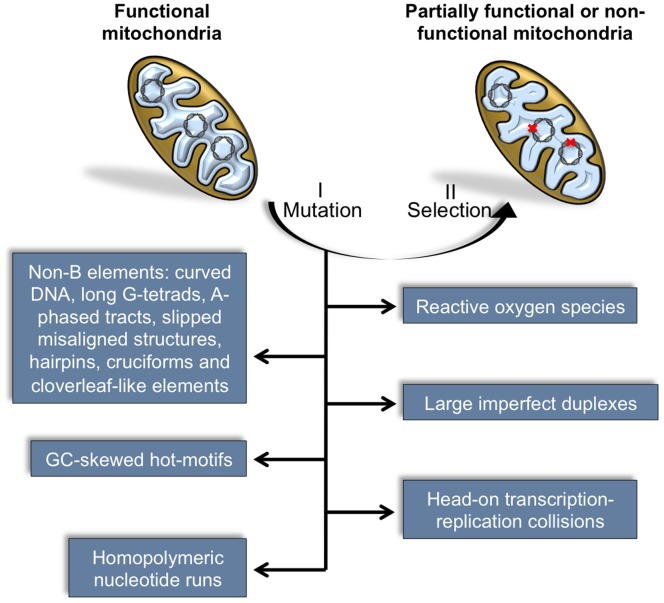
Diagram illustrating the sequence of events (I, II) capable of driving functional mitochondria to shift to a partially functional or non-functional state. The mutational events (I) may arise as a consequence of unusual DNA conformations, fragile motifs, exogenous factors, among others. These mutations will co-exist with the wild-type mtDNA in a heteroplasmic state, or eventually be selected (II) until a homoplasmic state is reached.

In conclusion, this study provides evidence supporting the idea that mtDNA instability arises from the concerted action of a *potpourri* of mechanisms, likely to provide adverse sequence contexts that favor genetic rearrangements (Fig. 6). Notably, our findings support the idea that local abrupt shifts in DNA composition provided by certain non-B structures or compositionally skewed motifs may represent important markers for genetic instability. Further research, will provide us with additional information on the YMMYMNNMMHM motif, and eventually extend it to a more general family of motifs.

## Supporting Information

Figure S1 **Three-dimensional reconstruction of the remaining 0.2 kb highly curved sequences highlighted in** **Fig. 2A** **.** The exact position corresponding to each curvature maximum is highlighted in red.
(TIF)

Figure S2 **Sequence logos for the most significant motifs found in regions flanking (±15 bp) deletion breakpoints using MEME and AlignACE.** MEME E values correspond to the expected number of motifs with equal or higher likelihood, with same width and number of occurrences in a set of random sequences of similar size and composition than the input sequence. The logos obtained were then trimmed using the STAMP tool, and the result is shown in Fig. 5A.
(TIF)

Table S1 **List of the 5′ and 3′ breakpoints from the 754 deletions analyzed in this study.** With the exception of two entries (marked with an asterisk), the list is similar to that recently published in [7]. Breakpoints were obtained from the references listed.
(DOCX)
