# Supplementary figures and images for: An Appraisal of Human Mitochondrial DNA Instability: New Insights into the Role of Non-Canonical DNA Structures and Sequence Motifs
Source: PLoS One. 2013 Mar 29;8(3):e59907. doi: 10.1371/journal.pone.0059907 (PMC3612095; doi:10.1371/journal.pone.0059907)

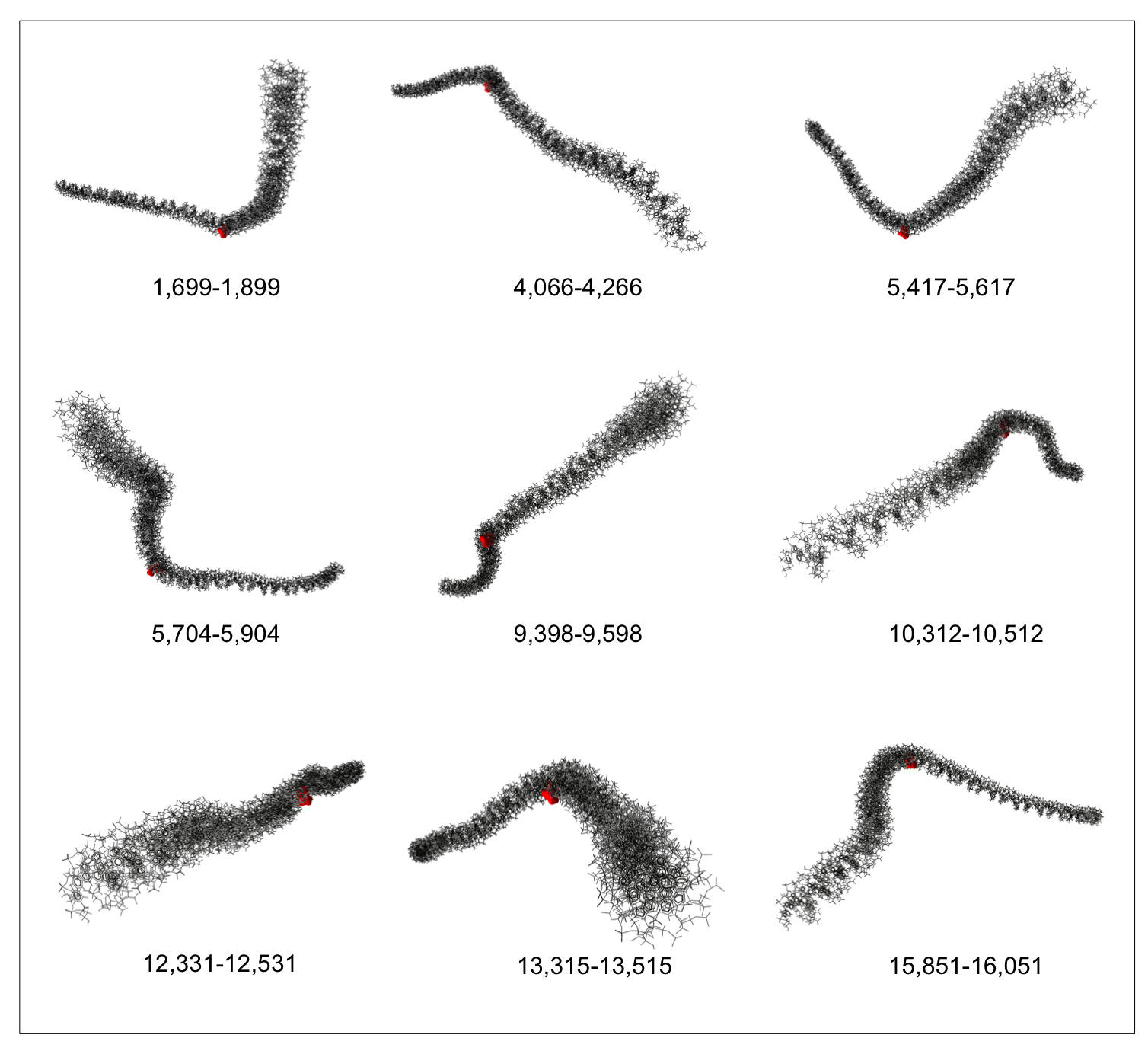

Supplement: Figure S1 — Three-dimensional reconstruction of the remaining 0.2 kb highly curved sequences highlighted in Fig. 2A . The exact position corresponding to each curvature maximum is highlighted in red. (TIF) [file pone.0059907.s001.tif]

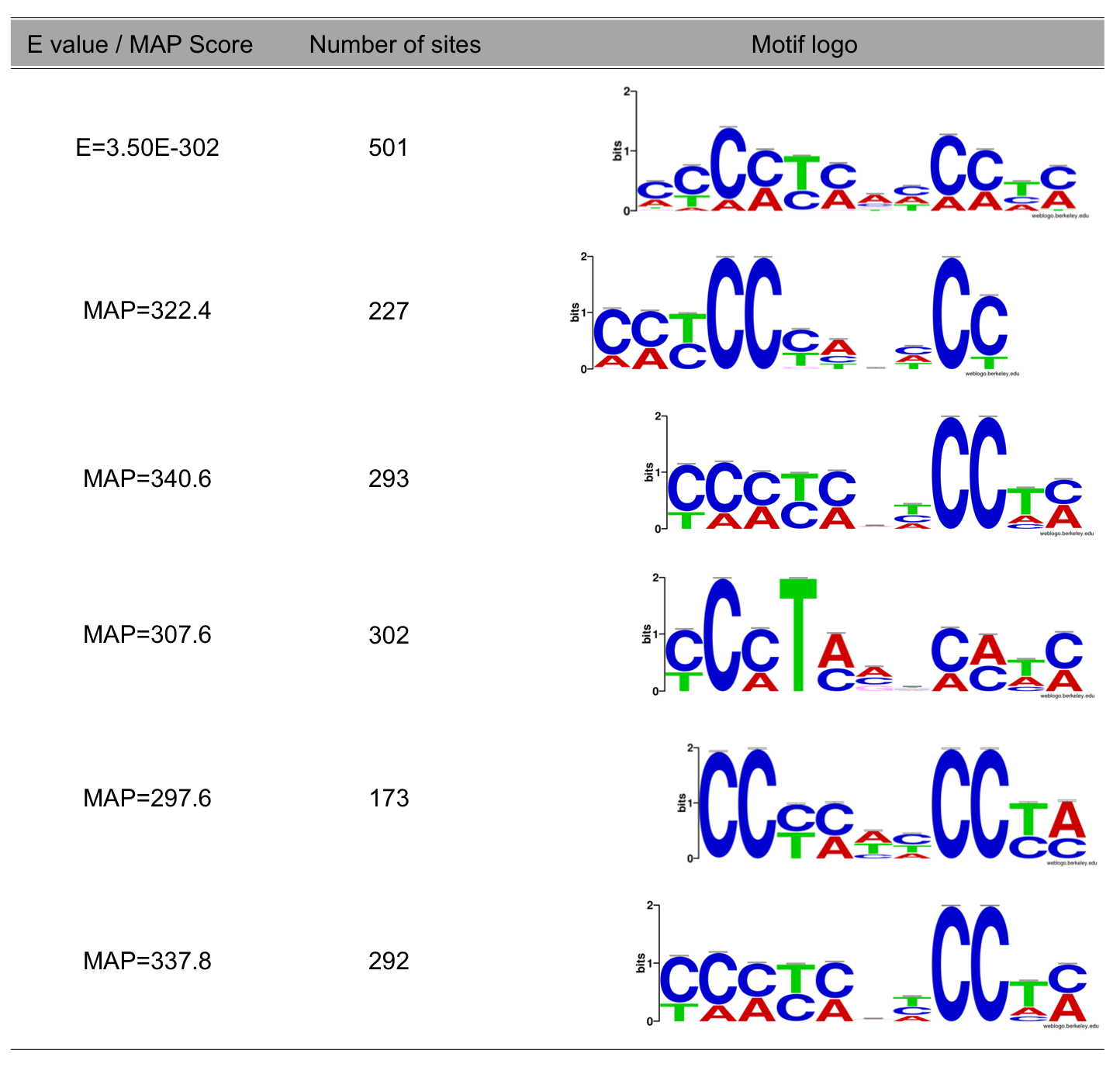

Supplement: Figure S2 — Sequence logos for the most significant motifs found in regions flanking (±15 bp) deletion breakpoints using MEME and AlignACE. MEME E values correspond to the expected number of motifs with equal or higher likelihood, with same width and number of occurrences in a set of random sequences of similar size and composition than the input sequence. The logos obtained were then trimmed using the STAMP tool, and the result is shown in Fig. 5A. (TIF) [file pone.0059907.s002.tif]
