# Supplementary material for: An Appraisal of Human Mitochondrial DNA Instability: New Insights into the Role of Non-Canonical DNA Structures and Sequence Motifs
Source: PLoS One. 2013 Mar 29;8(3):e59907. doi: 10.1371/journal.pone.0059907 (PMC3612095; doi:10.1371/journal.pone.0059907)
Supplement: Table S1 — List of the 5′ and 3′ breakpoints from the 754 deletions analyzed in this study. With the exception of two entries (marked with an asterisk), the list is similar to that recently published in [7]. Breakpoints were obtained from the references listed. (DOCX) [file pone.0059907.s003.docx]

**Supplementary Table S1**: List of the 5’ and 3’ breakpoints from the 754 deletions analyzed in this study. With the exception of two entries (marked with an asterisk), the list is similar to that recently presented in [23]. Breakpoints were obtained from the references shown below.

| Breakpoint positions (5’-3’) | | | | |
| --- | --- | --- | --- | --- |
| 105-112 | 3,270-16,034 | 5,787-13,924 | 6,423-14,344 | 7,128-14,006 |
| 299-349 | 3,272-15,068 | 5,787-15,576 | 6,428-15,270 | 7,182-14,566 |
| 306-357 | 3,272-16,034 | 5,787-16,035 | 6,429-14,101 | 7,185-14,120 |
| 463-12,080 | 3,311-15,741 | 5,787-16,071 | 6,437-14,077 | 7,188-14,335 |
| 471-5,152 | 3,326-3,591 | 5,787-16,072 | 6,453-14,288 | 7,193-14,587 |
| 478-5,160 | 3,326-13,923 | 5,787-16,073 | 6,464-14,874 | 7,193-14,596 |
| 488-8,643 | 3,393-16,072 | 5,788-13,239 | 6,466-14,135 | 7,201-14,809 |
| 493-14,242 | 3,418-3,432 | 5,788-15,448 | 6,469-15,586 | 7,204-13,903 |
| 504-5,443 | 3,432-16,071 | 5,788-16,069 | 6,476-13,777 | 7,212-14,261 |
| 537-4,431 | 3,486-16,068 | 5,788-16,071 | 6,476-14,146 | 7,215-16,072 |
| 544-13,768 | 3,549-15,440 | 5,789-16,075 | 6,504-13,805 | 7,215-16,073 |
| 547-4,443 | 3,549-16,072 | 5,793-12,767 | 6,521-16,071 | 7,218-16,071 |
| 585-4,443 | 3,571-15,540 | 5,795-13,920 | 6,527-15,055 | 7,235-16,072 |
| 633-16,262 | 3,575-15,968 | 5,798-16,072 | 6,533-16,075 | 7,235-16,074 |
| 708-13,767 | 3,591-11,519 | 5,803-16,072 | 6,537-13,838 | 7,265-16,035 |
| 898-16,116 | 3,614-12,987 | 5,836-12,662 | 6,551-13,852 | 7,265-16,071 |
| 1,417-15,219 | 3,692-16,068 | 5,987-13,537 | 6,555-14,345 | 7,267-16,077 |
| 1,491-5,206 | 3,742-16,074 | 6,003-15,441 | 6,572-12,304 | 7,268-16,077 |
| 1,491-5,323 | 3,826-16,071 | 6,019-6,025 | 6,577-15,154 | 7,277-12,300 |
| 1,672-16,071 | 3,963-11,432 | 6,023-14,424 | 6,585-15,144 | 7,288-15,900 |
| 1,691-13,925 | 4,048-15,949 | 6,062-16,070 | 6,587-14,285 | 7,289-14,943 |
| 1,741-14,502 | 4,069-14,306 | 6,074-9,179 | 6,601-16,284 | 7,322-13,464 |
| 1,753-13,101 | 4,141-16,073 | 6,076-13,799 | 6,625-16,074 | 7,322-15,239 |
| 1,771-16,072 | 4,351-16,071 | 6,084-13,808 | 6,634-9,925 | 7,323-15,056 |
| 1,840-5,451 | 4,376-16,078 | 6,124-15,893 | 6,750-16,072 | 7,323-15,435 |
| 1,906-16,072 | 4,398-14,822 | 6,128-15,434 | 6,753-16,075 | 7,329-14,531 |
| 1,907-16,079 | 4,458-16,074 | 6,172-13,057 | 6,759-15,865 | 7,331-15,946 |
| 1,989-14,366 | 4,469-13,923 | 6,173-16,078 | 6,790-15,918 | 7,337-14,931 |
| 2,059-16,071 | 4,486-16,075 | 6,175-14,162 | 6,836-14,380 | 7,338-14,598 |
| 2,068-16,074 | 4,882-16,068 | 6,189-16,072 | 6,843-14,388 | 7,347-14,245 |
| 2,333-16,075 | 5,042-14,552 | 6,203-14,258 | 6,851-14,816 | 7,350-16,071 |
| 2,333-16,078 | 5,173-15,607 | 6,215-10,976 | 6,862-13,061 | 7,360-15,276 |
| 2,334-16,073 | 5,195-16,071 | 6,218-13,447 | 6,919-13,564 | 7,366-14,060 |
| 2,540-16,047 | 5,328-16,076 | 6,226-13,447 | 6,927-16,072 | 7,367-12,063 |
| 2,781-16,071 | 5,330-16,077 | 6,238-14,103 | 6,935-15,447 | 7,367-16,072 |
| 2,818-16,078 | 5,440-13,030 | 6,310-15,611 | 6,940-15,451 | 7,370-16,072 |
| 3,167-14,154 | 5,561-13,405 | 6,320-11,272 | 6,961-16,071 | 7,370-16,073 |
| 3,173-14,161 | 5,671-16,071 | 6,326-13,989 | 7,057-16071 | 7,372-13,239 |
| 3,212-16,071 | 5,745-16,073 | 6,326-13,990 | 7,067-16,047 | 7,374-15,957 |
| 3,233-3,237 | 5,772-13,411 | 6,326-16,071 | 7,098-16,072 | 7,387-15,930 |
| 3,255-16,070 | 5,773-13,411 | 6,329-13,994 | 7,099-16,071 | 7,396-13,118 |
| 3,256-16,072 | 5,781-16,069 | 6,331-13,994 | 7,113-14,693 | 7,399-15,383 |
| 3,263-15,123 | 5,781-16,071 | 6,341-13,994 | 7,115-10,546 | 7,399-15,405 |
| 3,263-16,070 | 5,782-13,483 | 6,341-14,005 | 7,116-16,073 | 7,400-14,290 |
| 3,263-16,071 | 5,785-13,923 | 6,380-14,096 | 7,122-14,000 | 7,401-16,068 |
| 3,263-16,072 | 5,785-16,072 | 6,416-14,182 | 7,125-13,050 | 7,401-16,074 |
| 3,263-16,073 | 5,787-13,920 | 6,422-15,208 | 7,127-14,393 | 7,402-13,677 |

| Breakpoint positions (5’-3’) (cont.) | | | | |
| --- | --- | --- | --- | --- |
| 7,402-15,128 | 7,494-12,759* | 7,756-16,072 | 7,914-16,070 | 8,032-16,073 |
| 7,402-15,372 | 7,494-16,073 | 7,775-13,532 | 7,915-16,276 | 8,032-16,075 |
| 7,402-16,068 | 7,495-15,439 | 7,775-16,072 | 7,919-16,072 | 8,033-14,337 |
| 7,402-16,071 | 7,506-14,433 | 7,777-13,794 | 7,920-16,078 | 8,033-16,071 |
| 7,402-16,072 | 7,508-15,939 | 7,779-16,071 | 7,921-16,072 | 8,033-16,078 |
| 7,402-16,073 | 7,517-13,096 | 7,792-14,181 | 7,921-16,080 | 8,034-11,423 |
| 7,402-16,076 | 7,524-13,395 | 7,794-12,561 | 7,921-16,283 | 8,034-11,434 |
| 7,403-12,392 | 7,527-16,065 | 7,795-15,287 | 7,922-16,075 | 8,034-15,882 |
| 7,403-16,075 | 7,529-13,474 | 7,795-16,073 | 7,923-11,423 | 8,034-16,071 |
| 7,403-16,078 | 7,539-15,237 | 7,804-13,795 | 7,923-16,075 | 8,034-16,076 |
| 7,404-16,080 | 7,539-15,913 | 7,805-13,844 | 7,931-15,761 | 8,036-13,095 |
| 7,406-14,560 | 7,547-13,475 | 7,808-12,388 | 7,933-15,604 | 8,049-14,115 |
| 7,407-12,396 | 7,553-13,546 | 7,811-16,071 | 7,945-16,073 | 8,053-15,444 |
| 7,407-13,620 | 7,562-15,381 | 7,813-16,077 | 7,945-16,074 | 8,055-13,095 |
| 7,409-13,688 | 7,563-13,724 | 7,814-14,805 | 7,949-14,315 | 8,058-12,215 |
| 7,410-13,688 | 7,623-15,036 | 7,815-13,581 | 7,958-15,927 | 8,076-15,911 |
| 7,414-14,568 | 7,626-8,196 | 7,817-14,536 | 7,959-13,787 | 8,083-16,071 |
| 7,414-15,609 | 7,629-14,813 | 7,817-15,382 | 7,961-13,374 | 8,093-16,071 |
| 7,428-13,415 | 7,629-16,072 | 7,817-16,071 | 7,962-16,020 | 8,095-12,393 |
| 7,429-14,895 | 7,632-8,206 | 7,817-16,072 | 7,964-12,432 | 8,113-14,849 |
| 7,431-15,093 | 7,635-15,310 | 7,817-16,074 | 7,967-11,312 | 8,134-13,573 |
| 7,439-13,477 | 7,635-15,440 | 7,818-16,075 | 7,975-15,496 | 8,137-14,771 |
| 7,440-15,882 | 7,638-15,916 | 7,818-16,077 | 7,978-16,072 | 8,143-15,271 |
| 7,443-13,468 | 7,644-16,071 | 7,819-16,070 | 7,982-15,504 | 8,151-15,783 |
| 7,448-13,451 | 7,662-15,429 | 7,819-16,077 | 7,983-15,504 | 8,156-16,037 |
| 7,448-13,475 | 7,662-15,552 | 7,820-16,074 | 7,989-15,435 | 8,170-16,333 |
| 7,449-15,167 | 7,667-14,359 | 7,821-13,760 | 7,990-16,068 | 8,175-16,073 |
| 7,449-15,926 | 7,667-16,071 | 7,821-16,072 | 7,990-16,078 | 8,176-11,614 |
| 7,450-15,378 | 7,667-16,072 | 7,821-16,073 | 7,991-16,070 | 8,198-13,433 |
| 7,452-12,663 | 7,669-13,810 | 7,823-15,389 | 7,991-16,073 | 8,208-16,070 |
| 7,452-13,723 | 7,669-15,437 | 7,824-16,077 | 7,992-15,730 | 8,211-15,340 |
| 7,453-13,487 | 7,669-16,072 | 7,828-13,385 | 7,992-16,072 | 8,213-16,070 |
| 7,453-13,644 | 7,671-16,071 | 7,829-14,135 | 7,995-15,213 | 8,220-13,998 |
| 7,453-15,435 | 7,677-15,013 | 7,841-13,905 | 7,997-15,568 | 8,222-13,476 |
| 7,460-11,847 | 7,682-13,722 | 7,845-15,761 | 7,998-12,300 | 8,231-16,072 |
| 7,461-12,977 | 7,694-14,053 | 7,846-9,749 | 8,019-15,519 | 8,231-16,280 |
| 7,462-16,071 | 7,695-13,107 | 7,850-13,474 | 8,030-16,070 | 8,232-15,542 |
| 7,465-11,426 | 7,696-12,365 | 7,853-13,887 | 8,030-16,075 | 8,232-16,071 |
| 7,469-14,285 | 7,696-13,897 | 7,857-12,283 | 8,030-16,078 | 8,233-13,383 |
| 7,470-16,074 | 7,697-12,364 | 7,869-16,069 | 8,031-14,564 | 8,233-13,967 |
| 7,471-16,073 | 7,705-15,945 | 7,869-16,076 | 8,031-16,070 | 8,233-16,067 |
| 7,472-14,814 | 7,719-16,053 | 7,870-16,076 | 8,031-16,074 | 8,239-13,407 |
| 7,477-15,998 | 7,723-14,108 | 7,885-15,698 | 8,031-16,077 | 8,240-13,341 |
| 7,479-11,440 | 7,723-14,110 | 7,889-15,435 | 8,032-12,300 | 8,244-16,381 |
| 7,485-10,997 | 7,728-13,806 | 7,899-15,642 | 8,032-16,069 | 8,250-13,410 |
| 7,491-11,004 | 7,733-16,073 | 7,904-16,285 | 8,032-16,071 | 8,253-16,066 |
| 7,493-13,985 | 7,755-15,236 | 7,912-9,560 | 8,032-16,072 | 8,254-16,070 |

| Breakpoint positions (5’-3’) (cont.) | | | | |
| --- | --- | --- | --- | --- |
| 8,256-15,261 | 8,420-11,498 | 8,601-15,731 | 8,996-16,070 | 9,603-14,141 |
| 8,257-16,162 | 8,421-12,399 | 8,612-13,575 | 9,009-16,354 | 9,609-14,147 |
| 8,259-13,359 | 8,421-13,564 | 8,612-16,072 | 9,016-14,576 | 9,622-15,812 |
| 8,261-15,652 | 8,426-12,894 | 8,617-13,877 | 9,021-13,833 | 9,625-15,435 |
| 8,262-10,844 | 8,428-15,482 | 8,623-15,662 | 9,070-13,476 | 9,659-11,536 |
| 8,263-13,531 | 8,437-14,068 | 8,623-15,663 | 9,079-15,312 | 9,665-15,183 |
| 8,264-16,071 | 8,439-16,075 | 8,624-13,886 | 9,084-15,438 | 9,698-13,735 |
| 8,268-14,906 | 8,441-14,505 | 8,624-15,662 | 9,087-13,155 | 9,729-15,338 |
| 8,269-13,447 | 8,461-15,603 | 8,630-16,067 | 9,137-13,808 | 9,744-14,439 |
| 8,271-13,410 | 8,464-13,138 | 8,631-13,513 | 9,143-13,333 | 9,773-13,756 |
| 8,278-13,770 | 8,467-13,444 | 8,631-13,580 | 9,144-13,816 | 9,799-15,557 |
| 8,280-8,290 | 8,468-13,446 | 8,635-16,074 | 9,180-14,281 | 9,816-15,744 |
| 8,281-16,371 | 8,470-13,447 | 8,637-16,073 | 9,191-12,909 | 9,845-16,065 |
| 8,287-16,072 | 8,475-14,381 | 8,637-16,084 | 9,191-15,322 | 9,860-15,026 |
| 8,289-13,791 | 8,476-14,812 | 8,637-16,236 | 9,194-15,323 | 9,923-16,073 |
| 8,291-16,276 | 8,477-13,590 | 8,640-13,378 | 9,238-15,576 | 9,924-15,194 |
| 8,292-16,034 | 8,479-13,594 | 8,640-15,369 | 9,246-13,058 | 9,966-15,801 |
| 8,294-16,074 | 8,482-13,460 | 8,645-15,656 | 9,253-14,425 | 9,995-15,897 |
| 8,295-16,073 | 8,483-13,448 | 8,648-16,085 | 9,258-13,627 | 9,998-15,895 |
| 8,299-13,153 | 8,484-13,448 | 8,651-16,071 | 9,258-14,429 | 10,004-15,360 |
| 8,299-16,071 | 8,491-13,448 | 8,662-14,817 | 9,268-16,068 | 10,051-15,077 |
| 8,304-15,055 | 8,503-13,425 | 8,663-16,075 | 9,281-13,810 | 10,063-14,598 |
| 8,305-14,619 | 8,518-15,422 | 8,664-15,363 | 9,320-14,273 | 10,071-14,622 |
| 8,308-13,382 | 8,527-13,718 | 8,697-15,938 | 9,345-13,148 | 10,080-14,959 |
| 8,310-16,078 | 8,536-15,439 | 8,707-13,723 | 9,345-13,851 | 10,104-11,868 |
| 8,312-13,667 | 8,536-15,642 | 8,711-13,550 | 9,351-13,154 | 10,154-15,945 |
| 8,316-15,802 | 8,547-15,727 | 8,712-15,990 | 9,357-13,865 | 10,169-14,435 |
| 8,327-13,358 | 8,553-15,585 | 8,723-9,215 | 9,417-13,684 | 10,186-13,759 |
| 8,344-15,633 | 8,555-13,980 | 8,728-13,699 | 9,436-15,913 | 10,191-13,753 |
| 8,372-12,085 | 8,561-13,985 | 8,775-13,991 | 9,440-14,083 | 10,198-13,761 |
| 8,373-16,067 | 8,562-13,759 | 8,777-14,006 | 9,444-13,964 | 10,207-13,766 |
| 8,380-13,338 | 8,563-14,596 | 8,810-15,638 | 9,484-13,724 | 10,367-12,829 |
| 8,385-15,918 | 8,563-15,435 | 8,813-13,304 | 9,486-9,502 | 10,370-15,570 |
| 8,387-12,821 | 8,564-16,072 | 8,815-15,846 | 9,490-16,072 | 10,418-15,570 |
| 8,387-15,515 | 8,569-15,879 | 8,817-15,610 | 9,497-13,735 | 10,497-14,760 |
| 8,387-16,067 | 8,571-13,237 | 8,823-15,855 | 9,514-13,052 | 10,559-14,981 |
| 8,387-16,071 | 8,572-13,997 | 8,825-15,658 | 9,515-13,055 | 10,587-15,913 |
| 8,387-16,078 | 8,572-15,826 | 8,830-14,897 | 9,532-14,135 | 10,598-13,206 |
| 8,394-16,070 | 8,576-12,984 | 8,838-14,906 | 9,537-15,519 | 10,625-13,059 |
| 8,395-16,071 | 8,576-13,962 | 8,893-15,503 | 9,538-15,537 | 10,668-14,859 |
| 8,395-16,073 | 8,576-16,075 | 8,904-14,903 | 9,538-15,658 | 10,676-14,868 |
| 8,398-16,072 | 8,577-15,731 | 8,905-14,110 | 9,574-10,166 | 10,746-14,125 |
| 8,398-16,073 | 8,580-15,239 | 8,933-13,768 | 9,574-12,972 | 10,749-14,129 |
| 8,408-14,118 | 8,580-15,539 | 8,989-13,997 | 9,574-13,951 | 10,758-14,138 |
| 8,412-15,664 | 8,583-15,958 | 8,992-16,072 | 9,576-13,942 | 10,925-14,152 |
| 8,417-15,127 | 8,587-15,738 | 8,992-16,074 | 9,587-13,953 | 10,935-13,773 |
| 8,418-14,127 | 8,588-13,730 | 8,993-13,563 | 9,591-13,052 | 10,939-15,530 |

| Breakpoint positions (5’-3’) (cont.) | | | | |
| --- | --- | --- | --- | --- |
| 10,940-15,546 | 11,240-15,036 | 12,122-15,844 |  |  |
| 10,943-15,363 | 11,258-14,369 | 12,203-15,355 |  |  |
| 10,943-15,372 | 11,262-15,363* | 12,439-15,390 |  |  |
| 10,946-13,781 | 11,368-15,786 | 12,544-15,525 |  |  |
| 10,951-15,372 | 11,479-16,071 | 13,761-14,252 |  |  |
| 10,952-13,990 | 11,514-13,810 | 14,786-14,791 |  |  |
| 10,952-15,373 | 11,648-13,811 | 14,927-14,943 |  |  |
| 10,953-14,133 | 11,668-15,916 | 15,497-15,522 |  |  |
| 10,953-15,837 | 11,680-14,258 | 15,643-15,665 |  |  |
| 10,961-15,846 | 11,731-14,450 | 16,164-16,319 |  |  |
| 10,969-14,119 | 11,739-15,572 | 16,266-576 |  |  |
| 10,984-15,543 | 11,986-13,792 |  |  |  |
| 11,035-15,159 | 12,021-16,075 |  |  |  |
| 11,035-15,183 | 12,084-15,854 |  |  |  |
| 11,036-15,028 | 12,103-14,414 |  |  |  |
| 11,036-15,441 | 12,104-14,413 |  |  |  |
| 11,065-15,860 | 12,112-14,422 |  |  |  |
| 11,232-13,980 | 12,113-14,421 |  |  |  |
| 11,237-13,984 | 12,113-14,422 |  |  |  |

References:

1. Almeida E, Loureiro H, Almeida H, Machado MC, Cabral A, et al. (2007) Síndrome de Pearson. Caso Clínico. Acta Pediátrica Portuguesa 38:79-81.

2. Andreu AL, Hanna MG, Reichmann H, Bruno C, Penn AS, et al. (1999) Exercise intolerance due to mutations in the cytochrome b gene of mitochondrial DNA. N Engl J Med 341:1037-44.

3. Bai RK, Wong LJ (2005) Simultaneous detection and quantification of mitochondrial DNA deletion(s), depletion, and over-replication in patients with mitochondrial disease. J Mol Diagn 7:613-22.

4. Ballinger SW, Shoffner JM, Hedaya EV, Trounce I, Polak MA, et al. (1992) Maternally transmitted diabetes and deafness associated with a 10.4 kb mitochondrial DNA deletion. Nat Genet 1:11-5.

5. Bank C, Soulimane T, Schroder JM, Buse G, Zanssen S (2000) Multiple deletions of mtDNA remove the light strand origin of replication. Biochem Biophys Res Commun 279:595-601.

6. Baumer A, Zhang C, Linnane AW, Nagley P (1994) Age-related human mtDNA deletions: a heterogeneous set of deletions arising at a single pair of directly repeated sequences. Am J Hum Genet 54:618-30.

7. Behar DM, Blue-Smith J, Soria-Hernanz DF, Tzur S, Hadid Y, et al. (2008) A novel 154-bp deletion in the human mitochondrial DNA control region in healthy individuals. Hum Mutat 29:1387-91.

8. Bender A, Krishnan KJ, Morris CM, Taylor GA, Reeve AK, et al. (2006) High levels of mitochondrial DNA deletions in substantia nigra neurons in aging and Parkinson disease. Nat Genet 38:515-7.

9. Bene J, Nadasi E, Kosztolanyi G, Mehes K, Melegh B (2003) Congenital cataract as the first symptom of a neuromuscular disease caused by a novel single large-scale mitochondrial DNA deletion. Eur J Hum Genet 11:375-9.

10. Bi R, Zhang AM, Zhang W, Kong QP, Wu BL, et al. (2010) The acquisition of an inheritable 50-bp deletion in the human mtDNA control region does not affect the mtDNA copy number in peripheral blood cells. Hum Mutat 31:538-43.

11. Blakely EL, He L, Taylor RW, Chinnery PF, Lightowlers RN, et al. (2004) Mitochondrial DNA deletion in "identical" twin brothers. J Med Genet 41:e19.

12. Blok RB, Thorburn DR, Danks DM, Dahl HH (1995a) mtDNA deletion in a patient with symptoms of mitochondrial cytopathy but without ragged red fibers. Biochem Mol Med 56:26-30.

13. Blok RB, Thorburn DR, Thompson GN, Dahl HH (1995b) A topoisomerase II cleavage site is associated with a novel mitochondrial DNA deletion. Hum Genet 95:75-81.

14. Bodyak ND, Nekhaeva E, Wei JY, Khrapko K (2001) Quantification and sequencing of somatic deleted mtDNA in single cells: evidence for partially duplicated mtDNA in aged human tissues. Hum Mol Genet 10:17-24.

15. Brierley EJ, Johnson MA, Lightowlers RN, James OF, Turnbull DM (1998) Role of mitochondrial DNA mutations in human aging: implications for the central nervous system and muscle. Ann Neurol 43:217-23.

16. Bua E, Johnson J, Herbst A, Delong B, McKenzie D, et al. (2006) Mitochondrial DNA-deletion mutations accumulate intracellularly to detrimental levels in aged human skeletal muscle fibers. Am J Hum Genet 79:469-80.

17. Chabi B, Mousson de Camaret B, Duborjal H, Issartel JP, Stepien G (2003) Quantification of mitochondrial DNA deletion, depletion, and overreplication: application to diagnosis. Clin Chem 49:1309-17.

18. Comi GP, Bordoni A, Salani S, Franceschina L, Sciacco M, et al. (1998) Cytochrome c oxidase subunit I microdeletion in a patient with motor neuron disease. Ann Neurol 43:110-6.

19. Cormier V, Rotig A, Tardieu M, Colonna M, Saudubray JM, et al. (1991) Autosomal dominant deletions of the mitochondrial genome in a case of progressive encephalomyopathy. Am J Hum Genet 48:643-8.

20. Cormier-Daire V, Bonnefont JP, Rustin P, Maurage C, Ogler H, et al. (1994) Mitochondrial DNA rearrangements with onset as chronic diarrhea with villous atrophy. J Pediatr 124:63-70.

21. Corral-Debrinski M, Horton T, Lott MT, Shoffner JM, Beal MF, et al. (1992) Mitochondrial DNA deletions in human brain: regional variability and increase with advanced age. Nat Genet 2:324-9.

22. Coulter-Mackie MB, Applegarth DA, Toone JR, Gagnier L (1998) A protocol for detection of mitochondrial DNA deletions: characterization of a novel deletion. Clin Biochem 31:627-32.

23. Damas J, Carneiro J, Goncalves J, Stewart JB, Samuels DC, et al. (2012) Mitochondrial DNA deletions are associated with non-B DNA conformations. Nucleic Acids Res 40: 7606-7621.

24. Degoul F, Nelson I, Amselem S, Romero N, Obermaier-Kusser B, et al. (1991) Different mechanisms inferred from sequences of human mitochondrial DNA deletions in ocular myopathies. Nucleic Acids Res 19:493-6.

25. Eshaghian A, Vleugels RA, Canter JA, McDonald MA, Stasko T, et al. (2006) Mitochondrial DNA deletions serve as biomarkers of aging in the skin, but are typically absent in nonmelanoma skin cancers. J Invest Dermatol 126:336-44.

26. Fahn HJ, Wang LS, Kao SH, Chang SC, Huang MH, et al. (1998) Smoking-associated mitochondrial DNA mutations and lipid peroxidation in human lung tissues. Am J Respir Cell Mol Biol 19:901-9.

27. Ferlin T, Guironnet G, Barnoux MC, Dumoulin R, Stepien G, et al. (1997) Detection of mitochondrial DNA deletions by a screening procedure using the polymerase chain reaction. Mol Cell Biochem 174:221-5.

28. Fischel-Ghodsian N, Bohlman MC, Prezant TR, Graham JM, Jr., Cederbaum SD, et al. (1992) Deletion in blood mitochondrial DNA in Kearns-Sayre syndrome. Pediatr Res 31:557-60.

29. Fukushima K, Fiocchi C (2004) Paradoxical decrease of mitochondrial DNA deletions in epithelial cells of active ulcerative colitis patients. Am J Physiol Gastrointest Liver Physiol 286:G804-13.

30. Goios A, Nogueira C, Pereira C, Vilarinho L, Amorim A, et al. (2005) mtDNA single macrodeletions associated with myopathies: absence of haplogroup-related increased risk. J Inherit Metab Dis 28:769-78.

31. Harbottle A, Krishnan KJ, Birch-Machin MA (2004) Implications of using the ND1 gene as a control region for real-time PCR analysis of mitochondrial DNA deletions in human skin. J Invest Dermatol 122:1518-21.

32. Hayashi J, Ohta S, Kikuchi A, Takemitsu M, Goto Y, et al. (1991) Introduction of disease-related mitochondrial DNA deletions into HeLa cells lacking mitochondrial DNA results in mitochondrial dysfunction. Proc Natl Acad Sci U S A 88:10614-8.

33. Hinokio Y, Suzuki S, Komatu K, Ohtomo M, Onoda M, et al. (1995) A new mitochondrial DNA deletion associated with diabetic amyotrophy, diabetic myoatrophy and diabetic fatty liver. Muscle Nerve 3:S142-9.

34. Holt IJ, Harding AE, Morgan-Hughes JA (1989) Deletions of muscle mitochondrial DNA in mitochondrial myopathies: sequence analysis and possible mechanisms. Nucleic Acids Res 17:4465-9.

35. Horton TM, Petros JA, Heddi A, Shoffner J, Kaufman AE, et al. (1996) Novel mitochondrial DNA deletion found in a renal cell carcinoma. Genes Chromosomes Cancer 15:95-101.

36. Hsieh RH, Tsai NM, Au HK, Chang SJ, Wei YH, et al. (2002) Multiple rearrangements of mitochondrial DNA in unfertilized human oocytes. Fertil Steril 77:1012-7.

37. Jansson M, Darin N, Kyllerman M, Martinsson T, Wahlstrom J, et al. (2000) Multiple mitochondrial DNA deletions in hereditary inclusion body myopathy. Acta Neuropathol 100:23-8.

38. Johns DR, Cornblath DR (1991) Molecular insight into the asymmetric distribution of pathogenetic human mitochondrial DNA deletions. Biochem Biophys Res Commun 174:244-50.

39. Johns DR, Hurko O (1989) Preferential amplification and molecular characterization of junction sequences of a pathogenetic deletion in human mitochondrial DNA. Genomics 5:623-8.

40. Kajander OA, Rovio AT, Majamaa K, Poulton J, Spelbrink JN, et al. (2000) Human mtDNA sublimons resemble rearranged mitochondrial genoms found in pathological states. Hum Mol Genet 9:2821-35.

41. Kao SH, Chao HT, Wei YH (1998) Multiple deletions of mitochondrial DNA are associated with the decline of motility and fertility of human spermatozoa. Mol Hum Reprod 4:657-66.

42. Kapsa R, Thompson GN, Thorburn DR, Dahl HH, Marzuki S, et al. (1994) A novel mtDNA deletion in an infant with Pearson syndrome. J Inherit Metab Dis 17:521-6.

43. Keightley JA, Hoffbuhr KC, Burton MD, Salas VM, Johnston WS, et al. (1996) A microdeletion in cytochrome c oxidase (COX) subunit III associated with COX deficiency and recurrent myoglobinuria. Nat Genet 12:410-6.

44. Khrapko K, Bodyak N, Thilly WG, van Orsouw NJ, Zhang X, et al. (1999) Cell-by-cell scanning of whole mitochondrial genomes in aged human heart reveals a significant fraction of myocytes with clonally expanded deletions. Nucleic Acids Res 27:2434-41.

45. Kleinle S, Wiesmann U, Superti-Furga A, Krahenbuhl S, Boltshauser E, et al. (1997) Detection and characterization of mitochondrial DNA rearrangements in Pearson and Kearns-Sayre syndromes by long PCR. Hum Genet 100:643-50.

46. Klopstock T, Bischof F, Gerok K, Deuschl G, Seibel P, et al. (1995) 3.1-kb deletion of mitochondrial DNA in a patient with Kearns-Sayre syndrome. Acta Neuropathol 90:126-9.

47. Krauch G, Wilichowski E, Schmidt KG, Mayatepek E (2002) Pearson marrow-pancreas syndrome with worsening cardiac function caused by pleiotropic rearrangement of mitochondrial DNA. Am J Med Genet 110:57-61.

48. Kraytsberg Y, Kudryavtseva E, McKee AC, Geula C, Kowall NW, et al. (2006) Mitochondrial DNA deletions are abundant and cause functional impairment in aged human substantia nigra neurons. Nat Genet 38:518-20.

49. Larsson NG, Eiken HG, Boman H, Holme E, Oldfors A, et al. (1992) Lack of transmission of deleted mtDNA from a woman with Kearns-Sayre syndrome to her child. Am J Hum Genet 50:360-3.

50. Lebrecht D, Kokkori A, Ketelsen UP, Setzer B, Walker UA (2005) Tissue-specific mtDNA lesions and radical-associated mitochondrial dysfunction in human hearts exposed to doxorubicin. J Pathol 207:436-44.

51. Lee HC, Li SH, Lin JC, Wu CC, Yeh DC, et al. (2004) Somatic mutations in the D-loop and decrease in the copy number of mitochondrial DNA in human hepatocellular carcinoma. Mutat Res 547:71-8.

52. Lertrit P, Imsumran A, Karnkirawattana P, Devahasdin V, Sangruchi T, et al. (1999) A unique 3.5-kb deletion of the mitochondrial genome in Thai patients with Kearns-Sayre syndrome. Hum Genet 105:127-31.

53. Lezza AM, Cormio A, Gerardi P, Silvestri G, Servidei S, et al. (1997) Mitochondrial DNA deletions in oculopharyngeal muscular dystrophy. FEBS Lett 418:167-70.

54. Lim PS, Ma YS, Cheng YM, Chai H, Lee CF, et al. (2002) Mitochondrial DNA mutations and oxidative damage in skeletal muscle of patients with chronic uremia. J Biomed Sci 9:549-60.

55. Markaryan A, Nelson EG, Hinojosa R (2008) Detection of mitochondrial DNA deletions in the cochlea and its structural elements from archival human temporal bone tissue. Mutat Res 640:38-45.

56. Melov S, Schneider JA, Coskun PE, Bennett DA, Wallace DC (1999) Mitochondrial DNA rearrangements in aging human brain and in situ PCR of mtDNA. Neurobiol Aging 20:565-71.

57. Mita S, Rizzuto R, Moraes CT, Shanske S, Arnaudo E, et al. (1990) Recombination via flanking direct repeats is a major cause of large-scale deletions of human mitochondrial DNA. Nucleic Acids Res 18:561-7.

58. Mkaouar-Rebai E, Chamkha I, Kammoun T, Chabchoub I, Aloulou H, et al. (2010) A case of Kearns-Sayre syndrome with two novel deletions (9.768 and 7.253 kb) of the mtDNA associated with the common deletion in blood leukocytes, buccal mucosa and hair follicles. Mitochondrion 10:449-55.

59. Moraes CT, Ricci E, Petruzzella V, Shanske S, DiMauro S, et al. (1992) Molecular analysis of the muscle pathology associated with mitochondrial DNA deletions. Nat Genet 1:359-67.

60. Morikawa Y, Matsuura N, Kakudo K, Higuchi R, Koike M, et al. (1993) Pearson's marrow/pancreas syndrome: a histological and genetic study. Virchows Arch A Pathol Anat Histopathol 423:227-31.

61. Moslemi AR, Lindberg C, Oldfors A (1997) Analysis of multiple mitochondrial DNA deletions in inclusion body myositis. Hum Mutat 10:381-6.

62. Nakai A, Goto Y, Fujisawa K, Shigematsu Y, Kikawa Y, et al. (1994) Diffuse leukodystrophy with a large-scale mitochondrial DNA deletion. Lancet 343:1397-8.

63. Nakase H, Moraes CT, Rizzuto R, Lombes A, DiMauro S, et al. (1990) Transcription and translation of deleted mitochondrial genomes in Kearns-Sayre syndrome: implications for pathogenesis. Am J Hum Genet 46:418-27.

64. Nicholas A, Kraytsberg Y, Guo X, Khrapko K (2009) On the timing and the extent of clonal expansion of mtDNA deletions: evidence from single-molecule PCR. Exp Neurol 218:316-9.

65. Nishigaki Y, Marti R, Hirano M (2004) ND5 is a hot-spot for multiple atypical mitochondrial DNA deletions in mitochondrial neurogastrointestinal encephalomyopathy. Hum Mol Genet 13:91-101.

66. Norby S, Lestienne P, Nelson I, Nielsen IM, Schmalbruch H, et al. (1994) Juvenile Kearns-Sayre syndrome initially misdiagnosed as a psychosomatic disorder. J Med Genet 31:45-50.

67. Ota Y, Miyake Y, Awaya S, Kumagai T, Tanaka M, et al. (1994) Early retinal involvement in mitochondrial myopathy with mitochondrial DNA deletion. Retina 14:270-6.

68. Ota Y, Tanaka M, Sato W, Ohno K, Yamamoto T, et al. (1991) Detection of platelet mitochondrial DNA deletions in Kearns-Sayre syndrome. Invest Ophthalmol Vis Sci 32:2667-75.

69. Ozawa T (1995) Mechanism of somatic mitochondrial DNA mutations associated with age and diseases. Biochim Biophys Acta 1271:177-89.

70. Pak JW, Herbst A, Bua E, Gokey N, McKenzie D, et al. (2003) Mitochondrial DNA mutations as a fundamental mechanism in physiological declines associated with aging. Aging Cell 2:1-7.

71. Pang CY, Lee HC, Yang JH, Wei YH (1994) Human skin mitochondrial DNA deletions associated with light exposure. Arch Biochem Biophys 312:534-8.

72. Pineda M, Ormazabal A, Lopez-Gallardo E, Nascimento A, Solano A, et al. (2006) Cerebral folate deficiency and leukoencephalopathy caused by a mitochondrial DNA deletion. Ann Neurol 59:394-8.

73. Reeve AK, Krishnan KJ, Elson JL, Morris CM, Bender A, et al. (2008) Nature of mitochondrial DNA deletions in substantia nigra neurons. Am J Hum Genet 82:228-35.

74. Regan C (1999) Sequence characterization of breakpoint regions of human mitochondrial DNA deletions and possible mechanisms of formation. London, Ontario: The University of Western Ontario.

75. Reynier P, Figarella-Branger D, Serratrice G, Charvet B, Malthiery Y (1994) Association of deletion and homoplasmic point mutation of the mitochondrial DNA in an ocular myopathy. Biochem Biophys Res Commun 202:1606-11.

76. Rocher C, Letellier T, Copeland WC, Lestienne P (2002) Base composition at mtDNA boundaries suggests a DNA triple helix model for human mitochondrial DNA large-scale rearrangements. Mol Genet Metab 76:123-32.

77. Rogounovitch TI, Saenko VA, Shimizu-Yoshida Y, Abrosimov AY, Lushnikov EF, et al. (2002) Large deletions in mitochondrial DNA in radiation-associated human thyroid tumors. Cancer Res 62:7031-41.

78. Rotig A, Cormier V, Blanche S, Bonnefont JP, Ledeist F, et al. (1990) Pearson's marrow-pancreas syndrome. A multisystem mitochondrial disorder in infancy. J Clin Invest 86:1601-8.

79. Rotig A, Cormier V, Chatelain P, Francois R, Saudubray JM, et al. (1993) Deletion of mitochondrial DNA in a case of early-onset diabetes mellitus, optic atrophy, and deafness (Wolfram syndrome, MIM 222300). J Clin Invest 91:1095-8.

80. Rotig A, Cormier V, Koll F, Mize CE, Saudubray JM, et al. (1991) Site-specific deletions of the mitochondrial genome in the Pearson marrow-pancreas syndrome. Genomics 10:502-4.

81. Saiwaki T, Shiga K, Fukuyama R, Tsutsumi Y, Fushiki S (2000) A unique junctional palindromic sequence in mitochondrial DNA from a patient with progressive external ophthalmoplegia. Mol Pathol 53:333-5.

82. Schaefer AM, Blakely EL, Griffiths PG, Turnbull DM, Taylor RW (2005) Ophthalmoplegia due to mitochondrial DNA disease: the need for genetic diagnosis. Muscle Nerve 32:104-7.

83. Shanske S, Tang Y, Hirano M, Nishigaki Y, Tanji K, et al. (2002) Identical mitochondrial DNA deletion in a woman with ocular myopathy and in her son with pearson syndrome. Am J Hum Genet 71:679-83.

84. Solano A, Gamez J, Carod FJ, Pineda M, Playan A, et al. (2003) Characterisation of repeat and palindrome elements in patients harbouring single deletions of mitochondrial DNA. J Med Genet 40:e86.

85. Solano A, Russo G, Playan A, Parisi M, DiPietro M, et al. (2004) De Toni-Debre-Fanconi syndrome due to a palindrome-flanked deletion in mitochondrial DNA. Pediatr Nephrol 19:790-3.

86. Superti-Furga A, Schoenle E, Tuchschmid P, Caduff R, Sabato V, et al. (1993) Pearson bone marrow-pancreas syndrome with insulin-dependent diabetes, progressive renal tubulopathy, organic aciduria and elevated fetal haemoglobin caused by deletion and duplication of mitochondrial DNA. Eur J Pediatr 152:44-50.

87. Tanaka M, Sato W, Ohno K, Yamamoto T, Ozawa T (1989) Direct sequencing of deleted mitochondrial DNA in myopathic patients. Biochem Biophys Res Commun 164:156-63.

88. Tang Y, Schon EA, Wilichowski E, Vazquez-Memije ME, Davidson E, et al. (2000) Rearrangements of human mitochondrial DNA (mtDNA): new insights into the regulation of mtDNA copy number and gene expression. Mol Biol Cell 11:1471-85.

89. Tengan CH, Ferreiro-Barros C, Cardeal M, Fireman MA, Oliveira AS, et al. (2002) Frequency of duplications in the D-loop in patients with mitochondrial DNA deletions. Biochim Biophys Acta 1588:65-70.

90. Tengan CH, Kiyomoto BH, Rocha MS, Tavares VL, Gabbai AA, et al. (1998) Mitochondrial encephalomyopathy and hypoparathyroidism associated with a duplication and a deletion of mitochondrial deoxyribonucleic acid. J Clin Endocrinol Metab 83:125-9.

91. Vivekanandan P, Daniel H, Yeh MM, Torbenson M (2010) Mitochondrial mutations in hepatocellular carcinomas and fibrolamellar carcinomas. Mod Pathol 23:790-8.

92. Wanrooij S, Luoma P, van Goethem G, van Broeckhoven C, Suomalainen A, et al. (2004) Twinkle and POLG defects enhance age-dependent accumulation of mutations in the control region of mtDNA. Nucleic Acids Res 32:3053-64.

93. Yamamoto H, Tanaka M, Katayama M, Obayashi T, Nimura Y, et al. (1992) Significant existence of deleted mitochondrial DNA in cirrhotic liver surrounding hepatic tumor. Biochem Biophys Res Commun 182:913-20.
